# Effect of marital status on survival in glioblastoma multiforme by demographics, education, economic factors, and insurance status

**DOI:** 10.1002/cam4.1688

**Published:** 2018-07-15

**Authors:** Jun‐Chao Xie, Shuai Yang, Xue‐Yuan Liu, Yan‐Xin Zhao

**Affiliations:** ^1^ Department of Neurology Shanghai Tenth People's Hospital Tongji University School of Medicine Shanghai China; ^2^ Department of Ophthalmology Shanghai Tenth People's Hospital Tongji University School of Medicine Shanghai China

**Keywords:** cancer‐specific survival, glioblastoma, marital status, overall survival, SEER

## Abstract

The relationship between marital status and glioblastoma multiforme (GBM) has not been addressed in depth. Here, we aimed to investigate the association between marital status and survival in GBM. We searched the Surveillance, Epidemiology, and End Results (SEER) database and extracted the data of eligible patients diagnosed with GBM after 2004. Marital status was classified as married, divorced/separated, widowed, and single. A Kaplan‐Meier test was conducted to compare the survival curves of different groups. Multivariate Cox regression was performed to evaluate overall survival (OS) and cause‐specific survival (CSS) in different groups. Subgroup analysis was applied according to demographics, typical education and income levels in the locale, and insurance status. A total of 30 767 eligible patients were included. The median OS values were 9, 7, 3, 9 months in married, divorced/separated, widowed, and single patients, respectively. After adjustment for other covariates, married patients had better OS and CSS than other patients had. In addition to marital status, demographic factors, disease progression factors, local educational level, and insurance status were also associated with survival in GBM. Furthermore, subgroup analyses revealed the protective effect of marriage in most of the comparisons. Notably, the protective effect of marriage becomes more and more apparent as time goes on. The advantageous effect of marriage on GBM survival is especially prominent in patients who are male, older than 60 years of age, White, or living in middle‐income counties. In conclusion, marital status is an independent prognostic factor for GBM.

## 1 INTRODUCTION

Astrocytoma, the most common glioma in the central nervous system, is among the most aggressive tumors and has a poor prognosis. According to the most recent WHO 2016 classification, astrocytoma can be subdivided as follows: grade I, such as pilocytic astrocytoma and subependymal giant cell astrocytoma; grade II, low‐grade diffuse astrocytoma, such as fibrillary astrocytoma and gemistocytic astrocytoma; grade III, anaplastic astrocytoma; and grade IV, glioblastoma multiforme (GBM), a category that accounts for 60%‐70% of all astrocytomas with the worst prognosis. The prognosis of astrocytoma differs according to features such as age, disease stage, and histological type. GBM is the most malignant astrocytoma, with a median overall survival (OS) of approximately 12 months and a 5‐year OS of 4.8%‐5.4%,^1^, ^2^, ^3^ while patients suffering from anaplastic astrocytoma have a median OS of 38 months and a 5‐year OS of 25.9%‐41.1%.^1^, ^4^ A deeper understanding of the prognostic factors of GBM may provide new ideas for the prevention and management of this disease.

Marital status is a potential marker of mental status, lifestyle, and social and family support, and has a significant impact on the prognosis of patients with cardiovascular disease, cancer, or mental disorders.^5^, ^6^, ^7^ Using data from the US Surveillance, Epidemiology, and End Results (SEER) database, studies have determined that marital status is associated with overall and cancer‐specific survival in renal cancer, head and neck cancer, bladder cancer, and lung cancer.^8^, ^9^, ^10^, ^11^ However, whether modern marriage is beneficial for astrocytoma, especially GBM, is unknown. In this study, we aimed to investigate the relationship between marital status and the survival of patients with GBM, the most common and malignant astrocytoma, using the SEER database.

## 2 MATERIALS AND METHODS

### 2.1 Data source and selection criteria

All data were extracted from the SEER database with SEER*Stat software (version 8.3.5). The SEER database is an authoritative source of information on the incidence of cancer and the demographics, socioeconomic status, and survival of cancer patients in the United States; this database has been used for many high‐quality studies in the field of cancer research. We obtained permission to access the SEER research data files, with a reference number of 10540‐Nov 2017. The dataset used in this study was derived from the newest Incidence‐SEER 18 Regs Research Data + Hurricane Katrina Impacted Louisiana Cases, Nov 2017 Sub (1973–2015 varying). The data of patients diagnosed since 2004 and having “Site and Morphology Site recode ICD‐O‐3/WHO 2008” of “Brain and Other Nervous System” were extracted. Age, sex, race, marital status, year of diagnosis, vital status, cause of death, months of survival, laterality, surgery status, metastasis status, tumor size, SEER stage, percentage of the local population with at least a bachelors’ degree, local median household income, and insurance status were extracted from the SEER database for each patient.

Patients were included if they met both of the following criteria: (a) the histological type ICD‐O‐3 = 9440 (Glioblastoma, NOS); and (b) their marital status was married, divorced, separated, single, or widowed. Patients were excluded if they met any of the following criteria: (a) they were under 18 years old at diagnosis; (b) their cause of death was unknown; or (c) their survival time in months was unknown.

### 2.2 Variables and outcomes

The study variables included age, sex, race, year of diagnosis, vital status, cause of death, survival months, surgery status, metastasis status, tumor size, laterality, percentage of the local population with at least a bachelor's degree, local median household income, and insurance status. Marital status was classified into four groups: married, divorced or separated, single, and widowed. Patients were divided into four groups according to age: 49 years or younger, 50‐59 years, 60‐69 years, and ≥70 years. Patients diagnosed in different date ranges were also divided into three groups (2004‐2007, 2008‐2011, and 2012‐2015) to adjust for the survival difference caused by advances in the diagnosis and treatment of GBM with the passage of time. The registry site was divided into four groups based on geographic regions as follows: Northeast: Connecticut and New Jersey; South: Kentucky, Louisiana, Metropolitan Atlanta, Rural Georgia, Greater Georgia (excluding AT and RG); North Central: Metropolitan Detroit, Iowa; West: Hawaii, New Mexico, Seattle (Puget Sound), Utah, San Francisco‐Oakland SMSA, San Jose‐Monterey, Los Angeles, Greater California (excluding SF, LA, and SJ), Alaska. “County‐level median household income” and “% At least bachelor's degree” from the Census American Community Survey data were used to reflect the economic and educational status of the patients’ locales. These two variables were divided into quartiles: for median household income: quartile 1 (<US $50 600), quartile 2 (US $50 600‐58 580), quartile 3 (US $58 580‐70 930), and quartile 4 (>US $70 930); for % At least bachelor's degree: quartile 1 (<22.17%), quartile 2 (22.17%‐29.91%), quartile 3 (29.91%‐37.31%), and quartile 4 (>37.31%).

The primary outcomes were overall survival (OS) and GBM cancer‐specific survival (CSS). OS was defined as the survival time in months regardless of the cause of death. CSS was defined as the survival time in months from diagnosis to death due to GBM. Patients who were still alive at the end of the follow‐up or died of other causes were regarded as censored.

### 2.3 Statistical analysis

The baseline characteristics of patients with different marital status were compared using the chi‐squared test. The differences in OS and CSS were compared using the Kaplan‐Meier log‐rank test. Multivariate Cox regression analysis was applied to compare the OS and CSS in different marital status subgroups after adjusting for covariates, including sex, age, race, surgery status, metastasis status, tumor size, laterality, percentage of local residents with at least a bachelor's degree, local median household income, insurance status, year of diagnosis, and SEER stage.

In the propensity score matching (PSM) analysis, patients were divided into two groups: married and unmarried (the latter of which included divorced/separated, widowed, and single patients). The propensity score was determined with a binary logistic regression that included all the aforementioned covariates. A propensity score reflecting the probability of being married was then assigned to each patient. 1:1 PSM with no replacement was conducted using the nearest‐neighbor algorithm with a caliper width of 0.01.

Subgroup analyses were conducted to explore the association between marital status and GBM survival among patients differing in sex, age, race, registry site, diagnosis year, percentage of residents with at least a bachelor's degree in the region, median household income in the region, and insurance status. All *P* values were two‐sided. *P* values less than 0.05 were considered statistically significant. All analyses were performed using SPSS version 22.0 (SPSS Inc., Chicago, IL, USA).

## 3 RESULTS

### 3.1 Patient baseline characteristics

A total of 30 767 eligible GBM patients were included in this study. Among these patients, 20 076 (65.3%) were married, 2872 (9.3%) were divorced or separated, 3550 (11.5%) were widowed, and 4269 (13.9%) were single. The baseline characteristics of the eligible patients and the relationships between marital status and each variable were summarized in Table 1. Significant differences were noticed in almost all the comparisons. Most of the widowed patients were female (74.8%) and elderly (percentage aged ≥ 70 years: 78.2%). Married patients had the highest percentage of medical insurance coverage (69.0%), while single patients had the lowest percentage of medical insurance coverage (53.4%). Married patients also tended to have the highest surgery rate (76.5%), “no metastasis” rate (92.2%), localized SEER stage rate (78.1%), and percentage with tumor sizes ≤3 cm (15.1%).

**Table 1:** Baseline characteristics of eligible GBM patients in SEER database

Characteristic	Total (%)	Married (%)	Divorced/separated(%)	Widowed (%)	Single (%)	*P* value
	30767 (100)	20076 (65.3)	2872 (9.3)	3550 (11.5)	4269 (13.9)	
Sex(%)						
Male	17840 (58.0)	12897 (64.2)	1407 (49.0)	896 (25.2)	2640 (61.8)	<0.001
Female	12927 (42.0)	7179 (35.8)	1465 (51.0)	2654 (74.8)	1629 (38.2)	
Age						
≤49	4241 (13.8)	2586 (12.9)	351 (12.2)	22 (0.6)	1282 (30.0)	<0.001
50‐59	6953 (22.6)	4718 (23.5)	854 (29.7)	170 (4.8)	1211 (28.4)	
60‐69	8590 (27.9)	6037 (30.1)	956 (33.3)	581 (16.4)	1016 (23.8)	
≥70	10983 (35.7)	6735 (33.5)	711 (24.8)	2777 (78.2)	760 (17.8)	
Race						
White	27577 (89.6)	18253 (90.9)	2552 (88.9)	3214 (90.5)	3558 (83.3)	<0.001
Black	1692 (5.5)	799 (4.0)	222 (7.7)	187 (5.3)	484 (11.3)	
Others^a^	1441 (4.7)	993 (4.9)	94 (3.3)	141 (4.0)	213 (5.0)	
Unknown	57 (0.2)	31 (0.2)	4 (0.1)	8 (0.2)	14 (0.3)	
Registry sites						
Northeast	5028 (16.3)	3330 (16.6)	370 (12.9)	666 (18.8)	662 (15.5)	<0.001
South	6472 (21.0)	4277 (21.3)	635 (22.1)	793 (22.3)	767 (18.0)	
North Central	2996 (9.7)	1981 (9.9)	265 (9.2)	430 (12.1)	320 (7.5)	
West	16271 (52.9)	10488 (52.2)	1602 (55.8)	1661 (46.8)	2520 (59.0)	
Diagnosis year						
2004‐2007	9519 (30.9)	6248 (31.1)	890 (31.0)	1206 (34.0)	1175 (27.5)	<0.001
2008‐2011	10179 (33.1)	6649 (33.1)	932 (32.5)	1204 (33.9)	1394 (32.7)	
2012‐2015	11069 (36.0)	7179 (35.8)	1050 (36.6)	1140 (32.1)	1700 (39.8)	
At least a bachelors’ degree percent						
Quartile 1	7690 (25.0)	4970 (24.8)	740 (25.8)	960 (27.0)	1020 (23.9)	<0.001
Quartile 2	5160 (16.8)	3415 (17.0)	499 (17.4)	612 (17.2)	634 (14.9)	
Quartile 3	9805 (31.9)	6258 (31.2)	917 (31.9)	1104 (31.1)	1526 (35.7)	
Quartile 4	8110 (26.4)	5432 (27.1)	716 (24.9)	874 (24.6)	1088 (25.5)	
Unknown	2 (0.0)	1 (0.0)	0 (0.0)	0 (0.0)	1 (0.0)	
Median household income						
Quartile 1	7685 (25.0)	4976 (24.8)	779 (27.1)	957 (27.0)	973 (22.8)	<0.001
Quartile 2	7663 (24.9)	4807 (23.9)	732 (25.5)	846 (23.8)	1278 (29.8)	
Quartile 3	7705 (25.0)	5094 (25.4)	724 (25.2)	912 (25.7)	975 (22.8)	
Quartile 4	7712 (25.1)	5198 (25.9)	637 (22.2)	835 (23.5)	1042 (24.4)	
Unknown	2 (0.0)	1 (0.0)	0 (0.0)	0 (0.0)	1 (0.0)	
Insurance recode						
Insured	20059 (65.2)	13853 (69.0)	1634 (56.9)	2292 (64.6)	2280 (53.4)	<0.001
Any Medicaid	2453 (8.0)	1010 (5.0)	398 (13.9)	234 (6.6)	811 (19.0)	
Uninsured	754 (2.5)	371 (1.8)	111 (3.9)	39 (1.1)	233 (5.5)	
Unknown	7501 (24.4)	4842 (24.1)	729 (25.4)	985 (27.7)	945 (22.1)	
Laterality						
One side involvement	25099 (81.6)	16531 (82.3)	2316 (80.6)	2786 (78.5)	3466 (81.2)	<0.001
Bilateral involvement	457 (1.5)	295 (1.5)	43 (1.5)	49 (1.4)	70 (1.6)	
Paired site	284 (0.9)	169 (0.8)	30 (1.0)	36 (1.0)	49 (1.1)	
Not a paired site	4927 (16.0)	3081 (15.3)	483 (16.8)	679 (19.1)	684 (16.0)	
Surgery stratified						
Surgery performed	22835 (74.2)	15357 (76.5)	2155 (75.0)	2048 (57.7)	3275 (76.7)	<0.001
No surgical procedure of primary site	7841 (25.5)	4676 (23.3)	705 (24.5)	1473 (41.5)	987 (23.1)	
Unknown	91 (0.3)	43 (0.2)	12 (0.4)	29 (0.8)	7 (0.2)	
Metastasis						
No; none	28116 (91.4)	18513 (92.2)	2620 (91.2)	3081 (86.8)	3902 (91.4)	<0.001
Distant metastasis	392 (1.3)	252 (1.3)	39 (1.4)	46 (1.3)	55 (1.3)	
Unknown	2259 (7.3)	1311 (6.5)	213 (7.4)	423 (11.9)	312 (7.3)	
Tumor size						
≤3 cm	4421 (14.4)	3028 (15.1)	415 (14.4)	504 (14.2)	474 (11.1)	<0.001
3‐6 cm	15570 (50.6)	10269 (51.2)	1421 (49.5)	1781 (50.2)	2099 (49.2)	
>6 cm	5459 (17.7)	3430 (17.1)	541 (18.8)	579 (16.3)	909 (21.3)	
Unknown	5317 (17.3)	3349 (16.7)	495 (17.2)	686 (19.3)	787 (18.4)	
SEER Stage						
Localized	23795 (77.3)	15680 (78.1)	2170 (75.6)	2676 (75.4)	3269 (76.6)	<0.001
Regional	5584 (18.1)	3600 (17.9)	548 (19.1)	637 (17.9)	799 (18.7)	
Distant	450 (1.5)	292 (1.5)	41 (1.4)	50 (1.4)	67 (1.6)	
Unknown	938 (3.0)	504 (2.5)	113 (3.9)	187 (5.3)	134 (3.1)	

a Represents American Indian/AK Native, Asian/Pacific Islander).

### 3.2 Effect of marital status on overall and cause‐specific survival

**Figure 1:**
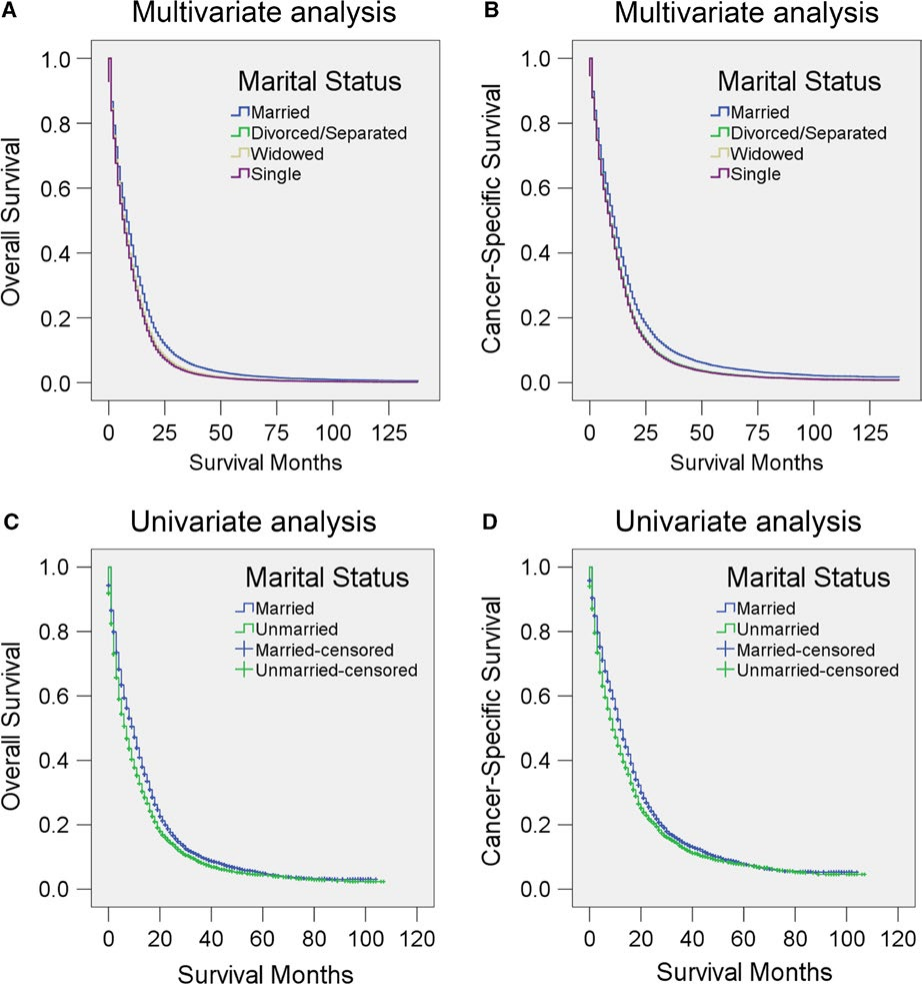
Survival curves for GBM patients according to marital status. Cox regression analyses for (A) overall survival and (B) cancer‐specific survival according to marital status. Logistic regression for (C) overall and (D) cancer‐specific survival according to marital status in a propensity score matching (PSM) analysis

A Kaplan‐Meier analysis was conducted to investigate the differences in OS and CSS across different groups defined by marital status and other variables (log‐rank test *P* < 0.001) (Tables 2, 3). The median OS was 9 months in the married group, 7 months in the divorced/separated group, 3 months in the widowed group, and 9 months in the single group (Table 2). After adjustment for age, sex, race, registry site, diagnose year, percentage local residents with of at least a bachelor's degree, local median household income, insurance status, laterality, surgery status, metastasis status, tumor size, and SEER stage, Cox regression indicated that, compared with married patients (as the reference group), divorced/separated (hazard ratio (HR): 1.184, 95% confidence interval (CI): 1.135, 1.235), widowed (HR: 1.176, 95% CI: 1.129, 1.225), and single (HR: 1.226, 95% CI: 1.180, 1.273) patients had poor OS (Table 2, Figure 1A). Regarding CSS, the median CSS values in the married, divorced/separated, widowed, and single groups were 12, 9, 5, and 12 months, respectively. Cox regression also indicated that married patients (as the reference group) had better CSS than divorced/separated (HR: 1.182, 95% CI: 1.127, 1.238), widowed (HR: 1.198, 95% CI: 1.143, 1.256), or single (HR: 1.200, 95% CI: 1.151, 1.251) patients after adjustment for other factors (Table 3, Figure 1B). In addition to the marital status of the patients, the data also indicated that sex, age, race, registry sites, diagnose year, percentage of local residents with at least a bachelor's degree, insurance status, laterality, surgery status, metastasis status, tumor size, and SEER stage are significantly associated with both OS and CSS in univariate analysis of these patients (Tables 2, 3). Moreover, after adjustment for all other covariates, all the aforementioned variables, except median household income and metastasis status, are still significantly associated with OS and CSS (Tables 2, 3). Female sex, younger age, Black or “other” race (American Indian/AK Native, Asian/Pacific Islander), residence in the Northeast (as represent by the registry site), more recent diagnosis year, higher local educational level (as reflected by percentage of residents with at least a bachelor's degree in the county), insurance, unilateral site, receipt of surgery, smaller tumor size, and localized SEER stage are significantly associated with better survival in GBM (Tables 2, 3).

**Table 2:** Univariate and multivariate analysis of overall survival (OS) for GBM patients

Variables	Median OS (month)	Univariate analysis		Multivariate analysis	
		Log‐rank χ^2^	*P* value	HR (95% CI)	*P* value
Sex					
Male	9	5.066	0.024	Reference	
Female	7			0.937 (0.914, 0.961)	<0.001
Age					
≤49	18	5422.897	<0.001	Reference	
50‐59	13			1.477 (1.414, 1.544)	<0.001
60‐69	9			1.977 (1.894, 2.063)	<0.001
≥70	3			3.358 (3.215, 3.508)	<0.001
Race					
White	8	67.655	<0.001	Reference	
Black	8			0.927 (0.879, 0.979)	0.006
Others	11			0.842 (0.792, 0.894)	<0.001
Registry sites					
Northeast	10	71.025	<0.001	Reference	
South	7			1.179 (1.123, 1.238)	<0.001
North Central	8			1.074 (1.017, 1.135)	0.010
West	8			1.123 (1.082, 1.165)	<0.001
Diagnosis year					
2004‐2007	7	69.196	<0.001	Reference	
2008‐2011	8			0.972 (0.932, 1.014)	0.191
2012‐2015	9			0.938 (0.898, 0.980)	0.004
Marital status					
Married	9	1298.106	<0.001	Reference	
Divorced/separated	7			1.184 (1.135, 1.235)	<0.001
Widowed	3			1.176 (1.129, 1.225)	<0.001
Single	9			1.226 (1.180, 1.273)	<0.001
At least a bachelors’ degree percent					
Quartile 1	6	193.957	<0.001	Reference	
Quartile 2	8			0.945 (0.906, 0.986)	0.009
Quartile 3	8			0.889 (0.853, 0.926)	<0.001
Quartile 4	10			0.824 (0.780, 0.870)	<0.001
Median household income					
Quartile 1	6	186.563	<0.001	Reference	
Quartile 2	8			0.973 (0.934, 1.013)	0.185
Quartile 3	8			1.038 (0.991, 1.088)	0.116
Quartile 4	10			0.993 (0.932, 1.058)	0.825
Insurance Recode					
Insured	9	87.561	<0.001	Reference	
Any Medicaid	8			1.169 (1.114, 1.226)	<0.001
Uninsured	11			1.150 (1.057, 1.252)	0.001
Laterality					
One side involvement	9	558.175	<0.001	Reference	
Bilateral involvement	3			1.250 (1.135, 1.378)	<0.001
Paired site	5			1.100 (0.968, 1.250)	0.144
Not a paired site	5			1.146 (1.108, 1.185)	<0.001
Surgery stratified					
Surgery performed	11	3970.905	<0.001	Reference	
No surgical procedure of primary site	3			1.819 (1.766, 1.874)	<0.001
Metastasis					
No; none	8	247.087	<0.001	Reference	
Distant metastasis	4			0.872 (0.648, 1.174)	0.367
Tumor size					
≤3 cm	10	178.857	<0.001	Reference	
3‐6 cm	9			1.131 (1.090, 1.173)	<0.001
>6 cm	6			1.307 (1.251, 1.365)	<0.001
SEER Stage					
Localized	9	764.782	<0.001	Reference	
Regional	5			1.336 (1.294, 1.381)	<0.001
Distant	4			1.622 (1.228, 2.142)	0.001

**Table 3:** Univariate and multivariate analysis of cancer‐specific survival (CSS) for GBM patients

Variables	Median CSS (month)	Univariate analysis		Multivariate analysis	
		HR (95% CI)	*P* value	HR (95% CI)	*P* value
Sex					
Male	11	9.723	0.002	Reference	
Female	10			0.964 (0.937, 0.992)	0.011
Age					
≤49	19	2651.251	<0.001	Reference	
50‐59	14			1.450 (1.384, 1.519)	<0.001
60‐69	11			1.814 (1.732, 1.899)	<0.001
≥70	5			2.689 (2.564, 2.821)	<0.001
Race					
White	11	42.535	<0.001	Reference	
Black	12			0.887 (0.834, 0.943)	<0.001
Others	13			0.870 (0.814, 0.929)	<0.001
Registry sites					
Northeast	13	75.410	<0.001	Reference	
South	10			1.178 (1.115, 1.245)	<0.001
North Central	11			1.076 (1.011, 1.144)	0.020
West	11			1.150 (1.103, 1.199)	<0.001
Diagnosis year					
2004‐2007	10	82.649	<0.001	Reference	
2008‐2011	11			0.992 (0.946, 1.040)	0.730
2012‐2015	12			0.948 (0.902, 0.995)	0.032
Marital status					
Married	12	805.604	<0.001	Reference	
Divorced/separated	9			1.182 (1.127, 1.238)	<0.001
Widowed	5			1.198 (1.143, 1.256)	<0.001
Single	12			1.200 (1.151, 1.251)	<0.001
At least a bachelors’ degree percentage					
Quartile 1	9	193.231	<0.001	Reference	
Quartile 2	10			0.925 (0.882, 0.970)	0.001
Quartile 3	11			0.883 (0.844, 0.924)	<0.001
Quartile 4	13			0.798 (0.750, 0.848)	<0.001
Median household income					
Quartile 1	9	164.682	<0.001	Reference	
Quartile 2	11			0.967 (0.924, 1.013)	0.154
Quartile 3	11			1.032 (0.980, 1.087)	0.237
Quartile 4	13			0.994 (0.926, 1.067)	0.868
Insurance Recode					
Insured	12	109.011	<0.001	Reference	
Any Medicaid	10			1.182 (1.121, 1.246)	<0.001
Uninsured	13			1.203 (1.099, 1.316)	<0.001
Laterality					
One side involvement	12	511.635	<0.001	Reference	
Bilateral involvement	5			1.281 (1.151, 1.425)	<0.001
Paired site	6			1.114 (0.967, 1.283)	0.134
Not a paired site	7			1.164 (1.121, 1.208)	<0.001
Surgery stratified					
Surgery performed	14	2981.314	<0.001	Reference	
No surgical procedure of primary site	4			1.838 (1.777, 1.900)	<0.001
Metastasis					
No; none	11	191.118	<0.001	Reference	
Distant metastasis	6			0.794 (0.577, 1.092)	0.157
Tumor size					
≤3 cm	14	168.518	<0.001	Reference	
3‐6 cm	12			1.151 (1.105, 1.200)	<0.001
>6 cm	9			1.335 (1.271, 1.402)	<0.001
SEER Stage					
Localized	12	730.439	<0.001	Reference	
Regional	6			1.363 (1.315, 1.414)	<0.001
Distant	5			1.790 (1.331, 2.409)	<0.001

To further confirm the finding that married patients survived longer and to minimize bias in the analysis, we conducted a PSM analysis. After the 1:1 PSM, a total of 10 598 patients (5299 married and 5299 unmarried) were included. The baseline variables are shown in Table 4. All the variables were clearly well matched (all *P* > 0.05). Logistic regression showed that the median OS and CSS, measured in months, were significantly longer in the married group than in the unmarried group (OS and CSS month in married group: 10 and 12 months; OS and CSS in unmarried groups: 7 and 9 months; Tables 5, 6, Figure 1C,D).

**Table 4:** Baseline characteristics for GBM patients after PSM

Characteristic	Total (%)	Married	Unmarried	*P* value
	10598 (100)	5299 (50)	5299 (50)	
Sex (%)				
Male	5222 (49.3)	2617 (49.4)	2605 (49.2)	0.816
Female	5376 (50.7)	2682 (50.6)	2694 (50.8)	
Age				
≤49	1508 (14.2)	766 (14.5)	742 (14.0)	0.749
50‐59	2265 (21.4)	1134 (21.4)	1131 (21.3)	
60‐69	2856 (26.9)	1405 (265)	1451 (27.4)	
≥70	3969 (37.5)	1994 (37.6)	1975 (37.3)	
Race				
White	9950 (93.9)	4976 (93.9)	4974 (93.9)	0.935
Black	648 (6.1)	323 (6.1)	325 (6.1)	
Registry sites				
Northeast	1466 (13.8)	735 (13.9)	731 (13.8)	0.324
South	2389 (22.5)	1233 (23.3)	1156 (21.8)	
North Central	1027 (9.7)	506 (9.5)	521 (9.8)	
West	5716 (53.9)	2825 (53.3)	2891 (54.6)	
Diagnosis year				
2004‐2007	1071 (10.1)	554 (10.5)	517 (9.8)	0.367
2008‐2011	4432 (41.8)	2226 (42.0)	2206 (41.6)	
2012‐2015	5095 (48.1)	2519 (47.5)	2576 (48.6)	
At least a bachelors’ degree percent				
Quartile 1	2857 (27.0)	1449 (27.3)	1408 (26.6)	0.135
Quartile 2	1779 (16.8)	904 (17.1)	875 (16.5)	
Quartile 3	3379 (31.9)	1633 (30.8)	1746 (32.9)	
Quartile 4	2583 (24.4)	1313 (24.8)	1270 (24.0)	
Median household income				
Quartile 1	2801 (26.4)	1386 (26.2)	1415 (26.7)	0.686
Quartile 2	2729 (25.8)	1350 (25.5)	1379 (26.0)	
Quartile 3	2654 (25.0)	1334 (25.2)	1320 (24.9)	
Quartile 4	2414 (22.8)	1229 (23.2)	1185 (22.4)	
Insurance Recode				
Insured	8915 (84.1)	4459 (84.1)	4456 (84.1)	0.852
Any Medicaid	1301 (12.3)	642 (12.1)	659 (12.4)	
Uninsured	382 (3.6)	198 (3.7)	184 (3.5)	
Laterality				
One side involvement	9106 (85.9)	4578 (86.4)	4528 (85.5)	0.599
Bilateral involvement	159 (1.5)	85 (1.6)	74 (1.4)	
Paired site	60 (0.6)	23 (0.4)	37 (0.7)	
Not a paired site	1273 (12.0)	613(11.6)	660 (12.5)	
Surgery stratified				
Surgery performed	8240 (77.8)	4116(77.7)	4124 (77.8)	0.934
No surgical procedure of primary site	2358 (22.2)	1183(22.3)	1175 (22.2)	
Metastasis				
No; none	10466 (98.8)	5230(98.7)	5236 (98.8)	0.817
Distant metastasis	132 (1.2)	69(1.3)	63 (1.2)	
Tumor size				
≤3 cm	1741 (16.4)	866(16.3)	875 (16.5)	0.431
3‐6 cm	6601 (62.3)	3298(62.2)	3303 (62.3)	
>6 cm	2256 (21.3)	1135(21.4)	1121 (21.2)	
SEER Stage				
Localized	8493 (80.1)	4225(79.7)	4268 (80.5)	0.173
Regional	1957 (18.5)	994(18.8)	963 (18.2)	
Distant	148 (1.4)	80(1.5)	68 (1.3)	

**Table 5:** Univariate analysis of overall survival (OS) for GBM patients after PSM

Variables	Median OS (month)	HR (95% CI)	Univariate analysis for OS	
			Log‐rank χ^2^	*P* value
Marital status				
Married	10	Reference	67.435	<0.001
Unmarried	7	1.183 (1.135, 1.233)		

**Table 6:** Univariate analysis of cancer‐specific survival (CSS) for GBM patients after PSM

Variables	Median CSS (month)	HR (95% CI)	Univariate analysis for CSS	
			Log‐rank χ^2^	*P* value
Marital status				
Married	12	Reference	45.654	<0.001
Unmarried	9	1.169 (1.115, 1.224)		

### 3.3 Subgroup analysis to evaluate the effect of marital status on CSS

**Figure 2:**
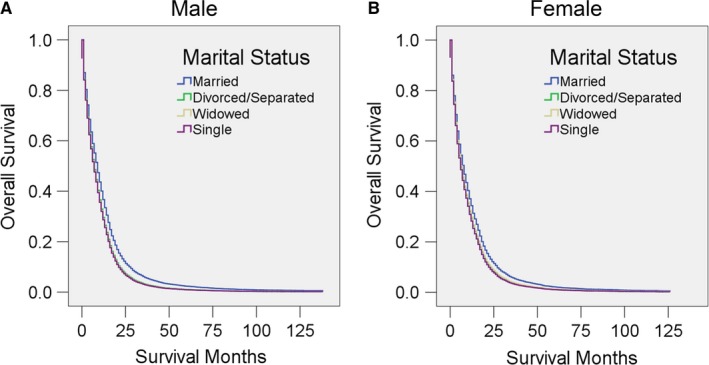
Survival curves for GBM patients according to marital status in different sexes. Cox regression analyses for overall survival in (A) male and (B) female patients

**Figure 3:**
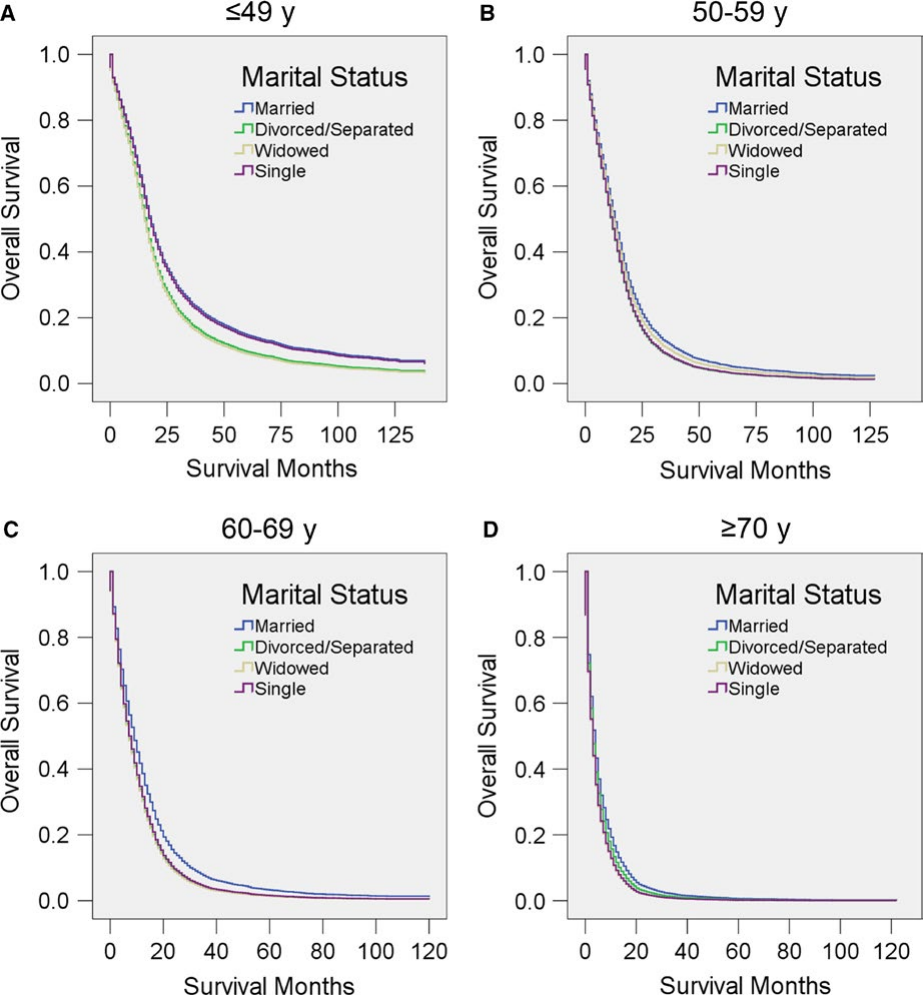
Survival curves for GBM patients according to marital status in different age range. Cox regression analyses for overall survival in patients aged A, ≤49, B 50‐59, C, 60‐69, and D, ≥70 years

We then performed subgroup analysis as the prognosis of GBM may vary according to demographic factors, educational environment, local economic status, year of diagnosis, and insurance status. Multivariate analysis showed that married patients of both sexes had better OS than other patients (all *P* < 0.001), although the HRs in unmarried male patients are all higher than those of their female counterparts (Figure 2, Table 7). We further analyzed OS and HR according to age range. Above the age of 60 years, married patients had better survival than unmarried patients. Surprisingly, widowed patients younger than 60 years old (*P* > 0.05) and single patients younger than 50 years old (*P* > 0.05) showed no differences from married patients of similar age (Figure 3, Table 8).

**Table 7:** Univariate and multivariate analysis for evaluating marital status on OS according to different sexes

Variables	Median OS (month)	Univariate analysis		Multivariate analysis	
		Log‐rank χ^2^	*P* value	HR (95% CI)	*P* value
Male					
Marital status		447.594	<0.001		
Married	9			Reference	
Divorced/separated	7			1.214 (1.145, 1.288)	<0.001
Widowed	3			1.234 (1.148, 1.326)	<0.001
Single	9			1.246 (1.187, 1.308)	<0.001
Female					
Marital status		844.693	<0.001		
Married	10			Reference	
Divorced/separated	7			1.151 (1.083, 1.223)	<0.001
Widowed	3			1.133 (1.075, 1.193)	<0.001
Single	8			1.184 (1.114, 1.257)	<0.001

**Table 8:** Univariate and multivariate analysis for evaluating marital status on OS according to different age ranges

Variables	Median OS (month)	Univariate analysis		Multivariate analysis	
		Log‐rank χ^2^	*P* value	HR (95% CI)	*P* value
≤49					
Marital status		12.091	0.007		
Married	18			Reference	
Divorced/separated	15			1.219 (1.077, 1.381)	0.002
Widowed	14			1.260 (0.790, 2.009)	0.332
Single	17			1.022 (0.943, 1.108)	0.595
50‐59					
Marital status		47.495	<0.001		
Married	14			Reference	
Divorced/separated	11			1.174 (1.082, 1.272)	<0.001
Widowed	14			1.072 (0.907, 1.266)	0.415
Single	10			1.164 (1.083, 1.251)	<0.001
60‐69					
Marital status		94.023	<0.001		
Married	10			Reference	
Divorced/separated	7			1.237 (1.149, 1.332)	<0.001
Widowed	6			1.258 (1.146, 1.381)	<0.001
Single	6			1.210 (1.124, 1.303)	<0.001
≥70					
Marital status		197.567	<0.001		
Married	4			Reference	
Divorced/separated	3			1.125 (1.038, 1.220)	0.004
Widowed	3			1.263 (1.202, 1.327)	<0.001
Single	3			1.246 (1.150, 1.348)	<0.001

**Figure 4:**
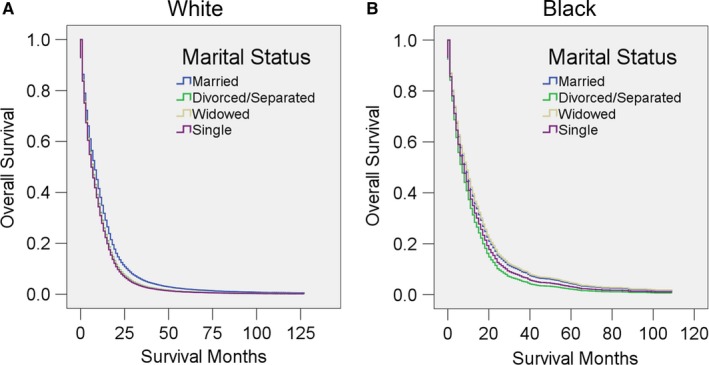
Survival curves for GBM patients according to marital status in different race. Cox regression analyses for overall survival in A, White and B, Black patients

Among White patients, married individuals had a better prognosis than any other marital status (all *P* < 0.001); Black patients, however, married individuals had no survival advantage over any other marital status except divorced/separated patients (*P* = 0.018) (Figure 4, Table 9).

**Table 9:** Univariate and multivariate analysis for evaluating marital status on OS according to different races

Variables	Median OS (month)	Univariate analysis		Multivariate analysis	
		Log‐rank χ^2^	*P* value	HR (95% CI)	*P* value
White					
Marital status		1235.754	<0.001		
Married	9			Reference	
Divorced/separated	7			1.175 (1.124, 1.229)	<0.001
Widowed	3			1.186 (1.136, 1.238)	<0.001
Single	9			1.216 (1.168, 1.267)	<0.001
Black					
Marital status		37.260	<0.001		
Married	10			Reference	
Divorced/separated	5			1.222 (1.035, 1.443)	0.018
Widowed	4			0.974 (0.804, 1.181)	0.790
Single	9			1.106 (0.967, 1.264)	0.140

**Figure 5:**
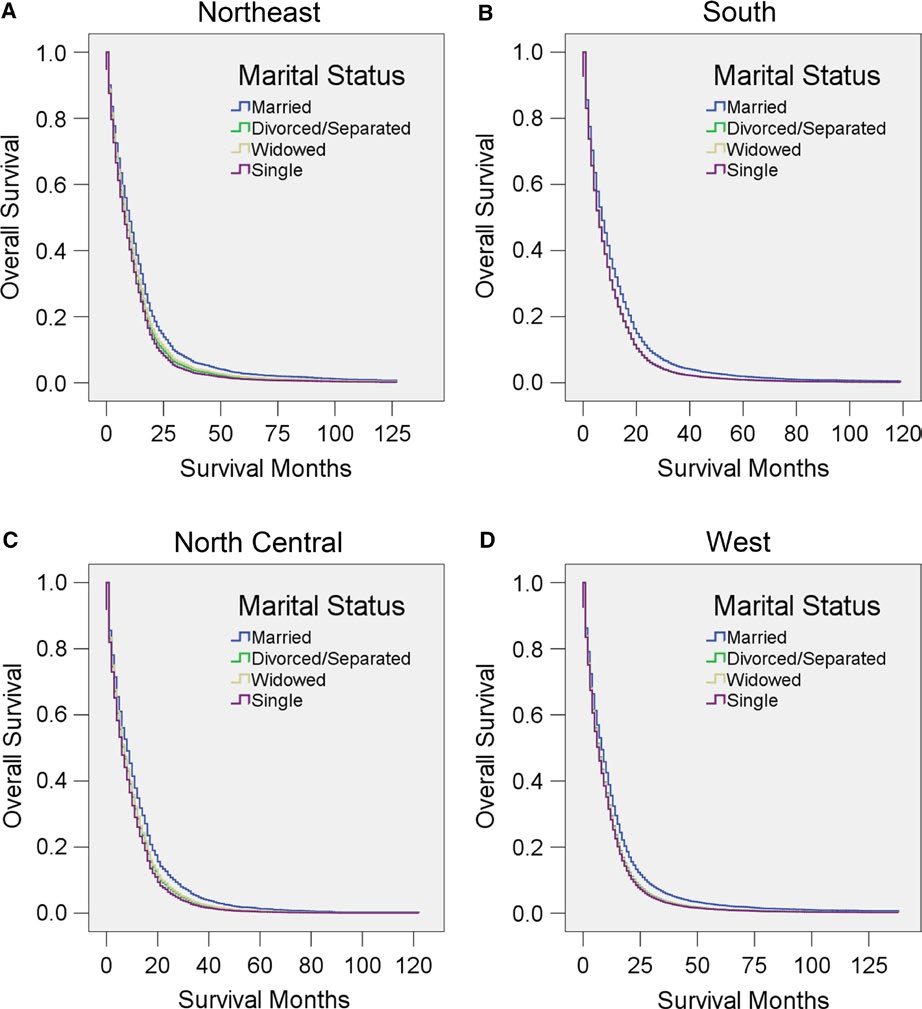
Survival curves for GBM patients according to marital status in different geographic regions. Cox regression analyses for overall survival in the A, Northeast, B, South, C, North Central, and D, West regions

We further stratified the patients into different geographic regions according to their registry sites: Northeast, South, North Central, and West. We found that marriage was associated with a better adjusted HR than any other marital status in all four regions (all *P* < 0.05; in some of the comparisons, *P* < 0.001), although in the North Central region, married patients have only a weakly significant advantage over divorced/separated and widowed patients (*P* = 0.017 or 0.016, respectively) (Figure 5, Table 10).

**Table 10:** Univariate and multivariate analysis for evaluating marital status on OS according to different geographic regions

Variables	Median OS (month)	Univariate analysis		Multivariate analysis	
		Log‐rank χ^2^	*P* value	HR (95% CI)	*P* value
Northeast					
Marital status		217.224	<0.001		
Married	11			Reference	
Divorced/separated	9			1.186 (1.054, 1.336)	0.005
Widowed	4			1.132 (1.027, 1.248)	0.012
Single	10			1.268 (1.150, 1.399)	<0.001
South					
Marital status		313.797	<0.001		
Married	8			Reference	
Divorced/separated	5			1.198 (1.095, 1.311)	<0.001
Widowed	3			1.192 (1.093, 1.300)	<0.001
Single	9			1.191 (1.091, 1.301)	<0.001
North Central					
Marital status		165.476	<0.001		
Married	9			Reference	
Divorced/separated	9			1.188 (1.032, 1.367)	0.017
Widowed	3			1.162 (1.028, 1.313)	0.016
Single	8			1.275 (1.112, 1.462)	0.001
West					
Marital status		623.824	<0.001		
Married	9			Reference	
Divorced/separated	7			1.182 (1.117, 1.251)	<0.001
Widowed	3			1.194 (1.125, 1.266)	<0.001
Single	9			1.222 (1.163, 1.284)	<0.001

**Figure 6:**
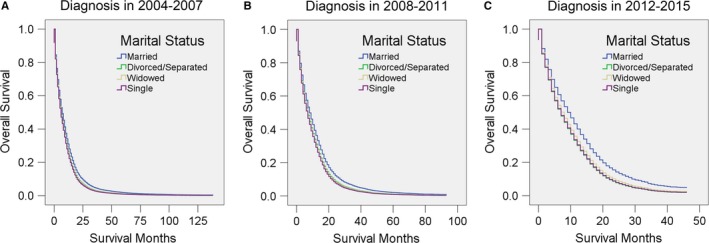
Survival curves for GBM patients according to marital status in different years of diagnosis. Cox regression analyses for the overall survival of patients diagnosed in A, 2004‐2007, B, 2008‐2011, and C, 2012‐2015

As emerging technologies have facilitated the diagnosis and treatment of GBM over time, we also stratified the patients by diagnosis year. Similarly, after adjustment for various factors, married patients had the best survival in all three subgroups (all *P* ≤ 0.001) (Figure 6, Table 11). Moreover, the adjusted HR of unmarried patients was higher in 2012‐2015 than in 2008‐2011, and higher in those periods than in 2004‐2007. These results indicate that the protective effect of marriage becomes more and more obvious as time goes on.

**Table 11:** Univariate and multivariate analysis for evaluating marital status on OS according to different diagnosis years

Variables	Median OS (month)	Univariate analysis		Multivariate analysis	
		Log‐rank χ^2^	*P* value	HR (95% CI)	*P* value
2004‐2007					
Marital status		405.376	<0.001		
Married	8			Reference	
Divorced/separated	7			1.127 (1.048, 1.211)	0.001
Widowed	3			1.143 (1.067, 1.225)	<0.001
Single	9			1.182 (1.106, 1.264)	<0.001
2008‐2011					
Marital status		457.196	<0.001		
Married	10			Reference	
Divorced/separated	8			1.152 (1.073, 1.237)	<0.001
Widowed	3			1.176 (1.098, 1.260)	<0.001
Single	8			1.216 (1.142, 1.295)	<0.001
2012‐2015					
Marital status		436.673	<0.001		
Married	10			Reference	
Divorced/separated	6			1.313 (1.216, 1.418)	<0.001
Widowed	3			1.225 (1.134, 1.322)	<0.001
Single	10			1.297 (1.211, 1.389)	<0.001

**Figure 7:**
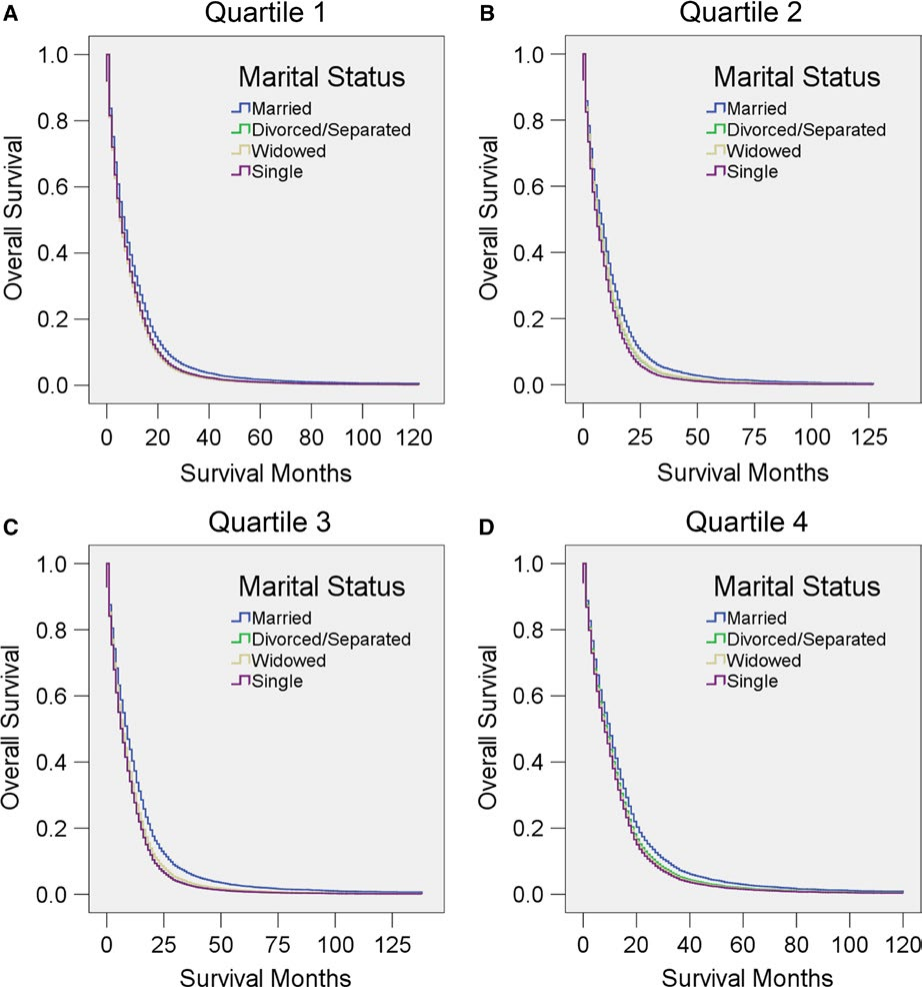
Survival curves for GBM patients according to marital status for different local education levels (defined as the percentage of the local population with at least a bachelors’ degree). Cox regression analyses for overall survival in A, quartile 1, B, quartile 2, C, quartile 3, and D, quartile 4

**Figure 8:**
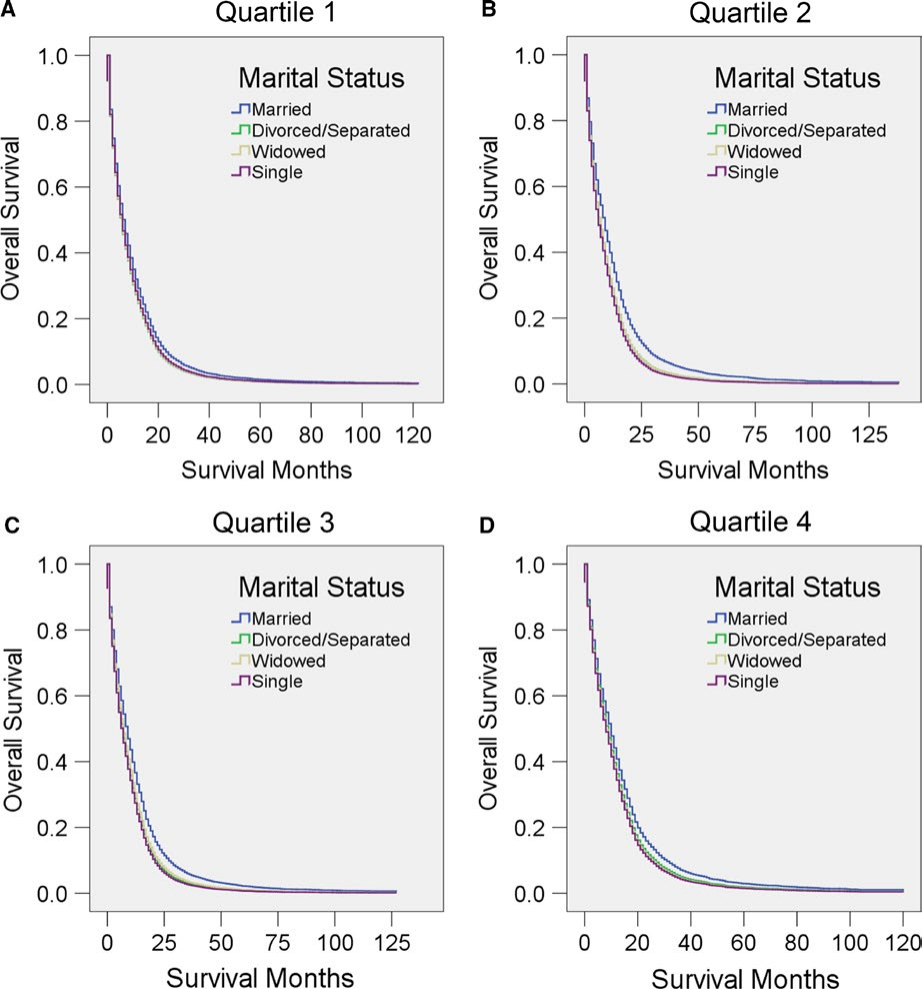
Survival curves for GBM patients according to marital status in different local economic levels (defined by median household income). Cox regression analyses for overall survival in A, quartile 1, B, quartile 2, C, quartile 3, and D, quartile 4

As local educational level and economic status may affect the treatment and outcome of GBM, we then stratified patients according to these two factors. It was clear that married patients showed significantly better survival than others after adjustment for other factors in all of these subgroups (all *P* < 0.05; in some of the comparisons, *P* < 0.001) (Figure 7, 8, Tables 12, 13). Notably, patients from the middle‐income counties (median household income in quartile 2 and quartile 3) seemed to benefit most from marriage, as the adjusted HR for unmarried patients was higher in these two income quartiles than in the others (Figure 8, Table 13).

**Table 12:** Univariate and multivariate analysis for evaluating marital status on OS according to different local education level (percentage of at least a bachelors’ degree)

Variables	Median OS (month)	Univariate analysis		Multivariate analysis	
		Log‐rank χ^2^	*P* value	HR (95% CI)	*P* value
Quartile 1					
Marital status		324.713	<0.001		
Married	7			Reference	
Divorced/separated	6			1.146 (1.055, 1.245)	0.001
Widowed	3			1.191 (1.100, 1.289)	<0.001
Single	8			1.149 (1.065, 1.240)	<0.001
Quartile 2					
Marital status		204.357	<0.001		
Married	9			Reference	
Divorced/separated	7			1.150 (1.039, 1.274)	0.007
Widowed	3			1.140 (1.034, 1.258)	0.009
Single	9			1.263 (1.145, 1.394)	<0.001
Quartile 3					
Marital status		450.370	<0.001		
Married	10			Reference	
Divorced/separated	7			1.301 (1.207, 1.403)	<0.001
Widowed	3			1.203 (1.117, 1.296)	<0.001
Single	8			1.302 (1.220, 1.389)	<0.001
Quartile 4					
Marital status		308.457	<0.001		
Married	11			Reference	
Divorced/separated	9			1.123 (1.031, 1.224)	0.008
Widowed	4			1.158 (1.067, 1.256)	<0.001
Single	11			1.189 (1.103, 1.281)	<0.001

**Table 13:** Univariate and multivariate analysis for evaluating marital status on OS according to different local economic level (median household income)

Variables	Median OS (month)	Univariate analysis		Multivariate analysis	
		Log‐rank χ^2^	*P* value	HR (95% CI)	*P* value
Quartile 1					
Marital status		300.849	<0.001		
Married	7			Reference	
Divorced/separated	6			1.136 (1.048, 1.232)	0.002
Widowed	3			1.129 (1.043, 1.222)	0.003
Single	8			1.104 (1.021, 1.194)	0.013
Quartile 2					
Marital status		347.733	<0.001		
Married	9			Reference	
Divorced/separated	7			1.240 (1.141, 1.348)	<0.001
Widowed	3			1.242 (1.143, 1.351)	<0.001
Single	8			1.325 (1.235, 1.422)	<0.001
Quartile 3					
Marital status		332.606	<0.001		
Married	10			Reference	
Divorced/separated	8			1.239 (1.139, 1.348)	<0.001
Widowed	3			1.183 (1.091, 1.283)	<0.001
Single	9			1.289 (1.190, 1.396)	<0.001
Quartile 4					
Marital status		307.507	<0.001		
Married	11			Reference	
Divorced/separated	9			1.130 (1.032, 1.238)	0.008
Widowed	3			1.170 (1.076, 1.273)	<0.001
Single	11			1.192 (1.105, 1.286)	<0.001

**Figure 9:**
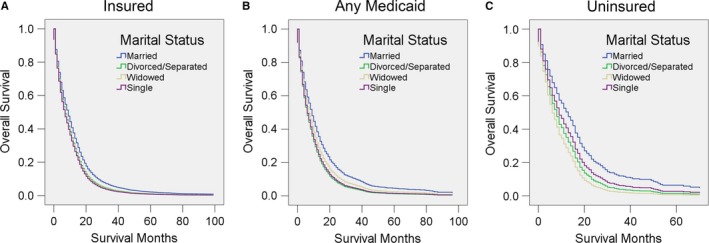
Survival curves for GBM patients according to marital status in different insurance statuses. Cox regression analyses for overall survival in A, insured patients, B, patients with any Medicaid coverage, and C, uninsured patients

In addition, we stratified patients by insurance status. Consistent with previous results, married patients had a survival advantage in almost all the comparisons. Interestingly, when compared with divorced/separated or widowed patients, married patients with no insurance seemed to benefit more from their marriage (Figure 9, Table 14).

**Table 14:** Univariate and multivariate analysis for evaluating marital status on OS according to different medical insurance status

Variables	Median OS (month)	Univariate analysis		Multivariate analysis	
		Log‐rank χ^2^	*P* value	HR (95% CI)	*P* value
Insured					
Marital status		886.398	<0.001		
Married	10			Reference	
Divorced/separated	8			1.178 (1.114, 1.246)	<0.001
Widowed	3			1.209 (1.148, 1.272)	<0.001
Single	9			1.245 (1.184, 1.309)	<0.001
Any Medicaid					
Marital status		53.930	<0.001		
Married	9			Reference	
Divorced/separated	6			1.398 (1.226, 1.595)	<0.001
Widowed	4			1.196 (1.013, 1.412)	0.035
Single	7			1.345 (1.204, 1.502)	<0.001
Uninsured					
Marital status		32.219	<0.001		
Married	13			Reference	
Divorced/separated	6			1.523 (1.188, 1.953)	0.001
Widowed	4			1.811 (1.230, 2.665)	0.003
Single	11			1.306 (1.058, 1.612)	0.013

## 4 DISCUSSION

In this study, we discovered that married patients had a better prognosis than those who were not married, even after adjusting for other variables. Our result is consistent with an observation by Chang et al.,^12^ which also indicated that marriage is beneficial for the prognosis of patients with GBM. Herein, we update their findings in more recently diagnosed patients (since 2004). Furthermore, we included many more patients and examined two additional categories of marital status. Our study revealed that single patients, compared with the other categories of marital status, had the highest adjusted HR for both OS and CSS. The relatively long survival of single patients in Kaplan‐Meier analysis is partially due to their relatively young age. In addition, to avoid the bias caused by advances in diagnosis and treatment technology with the passage of time, we included only those patients who were diagnosed after 2004 and conducted a subgroup analysis that stratified diagnosis year into three ranges. This subgroup analysis also indicated that married patients had a better CSS than others, while single patients had the worst OS before 2012. This trend is also observed in non–small‐cell lung cancer.^11^ Interestingly, after 2012, the adjusted OS of divorced/separated patients was even worse than that of single patients. Our result also indicated that the protective effect of marriage becomes stronger as time goes on. A possible explanation is that people have become increasingly serious about marriage in recent years, which contributes to the quality of marriage and the amount of mental and physical support it brings.

Our results in this study are consistent to some extent with the previous findings in other types of cancer,^7^, ^8^, ^9^, ^10^, ^11^, ^13^ which indicated that married patients had a survival advantage over other patients. However, the underlying mechanisms are not fully understood. There are several possible reasons. First, marriage may have provided economic support, which enables the patients to receive an improved quality of treatment. Second, previous studies have indicated that mental disorders caused by cancer not only decrease individuals the willingness to adhere to treatment, but also directly increase overall cancer mortality.^14^, ^15^ Marriage provides the patients with strong mental support from their partners or families, which helps them release or share the pain and the depressed or anxious mood caused by the disease.^16^, ^17^, ^18^ Third, life habits, such as smoking, drinking, and diet control, can be strongly influenced by marriage. Fourth, married patients might be diagnosed at an earlier stage, which would also partially contribute to better survival. Fifth, marriage influences the function of body physiologically, partially through modulating the level of endocrinal hormones that affect the prognosis of GBM.^19^, ^20^, ^21^ In addition, the offspring of married couples provide additional support that is often unavailable to single patients.^22^ The influence of offspring also partially explains why the survival of divorced/separated and widowed patients is better than that of single patients.

In addition to multivariate Cox regression, PSM has been proposed as another method to reduce the impact of patient selection bias on observational data and mimic randomized controlled trials.^23^, ^24^ PSM has been widely used in several areas of medical research including studies to assess factors associated with cancer survival.^25^, ^26^, ^27^, ^28^, ^29^ In our study, after 1:1 matching, married patients showed survival advantage over unmarried patients.

Furthermore, our study indicated that male GBM patients benefit more from marriage than female patients do, which is also consistent with findings in other kinds of cancers as reported by Aizer et al.^7^ There are several potential reasons for this discrepancy. First, marriage may provide more mental support to males than to females. Second, males tend to have more bad habits such as smoking and drinking than females have, meaning that males benefit more than females from the lifestyle change caused by marriage. In addition, in married female patients, pregnancy accelerates the progression of astrocytoma.^30^, ^31^, ^32^

Surprisingly, among people aged ≤59 years, widowed patients showed no difference in OS compared with married patients. A potential partial explanation is that young widowed patients can still obtain some of the family, social, or economic support that they previously accessed through their partners. For example, widows and widowers they can obtain mental support from their children or parents‐in‐law.^22^ Furthermore, widowed patients may have a better financial situation or insurance status than single patients or divorced/separated patients because the former can rely on inheritances from their partners. Furthermore, the adjusted survival of young single patients (aged ≤ 49 years) showed no difference from that of married patients. This result indicated that older patients benefit more from marriage. Single young adults care more about their health than older patients do and may comply effectively with more aggressive treatment 33 making them less dependent on marriage for GBM survival.

Our results indicated that race also influenced the GBM survival, with marriage improving survival in White but not in Black patients. Similar results were observed in patients with renal cancer.^34^ Prior studies have indicated that the social and mental support provided by marriage is different across different races. The underlying mechanisms of this difference may need further investigation.

Our results indicated that local educational level is a protective factor in GBM, while local economic status has no association with the prognosis of GBM. These findings imply that the former factor but not the latter may provide support for the patients. This result is consistent with a previous study indicating that Swedish cancer patients with higher educational levels tend to have better survival.^35^ Patients from region with higher educational levels may care more about their health and be willing to receive more earlier and more effective interventions.^35^ When the patients were stratified according to local educational level or economic status, married patients showed significantly better adjusted survival than others in all of the subgroups. Moreover, patients from the middle‐income counties seemed to benefit more from marriage than residents of upper‐ or lower‐income counties did. This result is interesting, but the underlying mechanism needs further research.

Our results indicated that insurance status is also a protective factor for GBM patients. When the patients were stratified according to insurance status, marriage was associated with an OS advantage compared with all three of the other marital statuses. Interestingly, compared with other patients, married patients seemed to benefit more from their marriage the less insurance they had. These results suggest to us that insurance is especially important for unmarried patients.

This study has certain limitations. The quality of marriage, which may also influence the outcomes of patients, could not be defined clearly. In addition, some variables, such as SEER stage, insurance status, and race, are unknown in a portion of patients.

Despite the limitations mentioned above, we demonstrated an intriguing association between marital status and the survival of GBM patients in a large population. In conclusion, married patients had a better prognosis than others. Furthermore, the adjusted survival rate of single patients is even worse than that of either widowed or divorced/separated patients. These differences may be caused by psychological, physiological, social, or economic factors that arise from marital status. Clinical staff should aware of the relatively poor prognosis for unmarried patients, especially single patients.

## CONFLICT OF INTEREST

None.

## ETHICAL APPROVAL

The informed consent was not required in this study, because personal identifying information was not included in the SEER database.
